# Predictors for the severity of acute aquaporin-4 immunoglobulin G-seropositive neuromyelitis optica spectrum disorders: a prospective cohort study

**DOI:** 10.3389/fimmu.2026.1714655

**Published:** 2026-07-13

**Authors:** Yibo Zhan, Min Zhao, Jiahui Zhang, Jiayin Li, Zequan Zheng, Shanshan Fu, Boyan Pan, Chang Zhou, Hui Xia, Guixian Chen, Haoyou Xu, Yuanqi Zhao

**Affiliations:** 1 The Second Clinical College of Guangzhou University of Chinese Medicine, Guangzhou, China; 2 Guangdong Provincial Second Hospital of Traditional Chinese Medicine, Guangzhou, China; 3 Department of Neurology, The Second Affiliated Hospital of Guangzhou University of Chinese Medicine, Guangdong Provincial Hospital of Chinese Medicine, Guangzhou, China

**Keywords:** aquaporin-4, complement, expanded disability status scale score, neuromyelitis optica spectrum disorder, NLR

## Abstract

**Objective:**

The study aimed to investigate risk factors for disease severity in acute aquaporin-4 (AQP4) -IgG-seropositive neuromyelitis optica spectrum disorders (NMOSD) patients.

**Methods:**

This prospective cohort study enrolled 103 serum Aquaporin-4 Immunoglobulin G (AQP4-IgG)-positive NMOSD patients from the Department of Neurology, Guangdong Provincial Hospital of Chinese Medicine, between April 2022 and June 2025. Among them, 60 cases were in the acute phase and 43 in the remission phase. Additionally, 25 age-and sex-matched healthy controls (HCs) were included. Clinical characteristics of all participants were evaluated, and blood samples were collected for laboratory testing. The Expanded Disability Status Scale (EDSS) score was used to assess the severity of the disease. Receiver operating characteristic (ROC) curve analysis was performed using EDSS ≥ 4 as the definition of severe disability.

**Results:**

Our cohort study revealed significant differences (P < 0.05) in white blood cell (WBC) count, neutrophil (NEUT) count, neutrophil-to-lymphocyte ratio (NLR), monocyte-to-lymphocyte ratio (MLR), systemic immune inflammation index (SII), systemic inflammation response index (SIRI), and serum complement levels among patients in the acute phase, those in the remission phase, and HCs. Multivariate regression analysis demonstrated that age (p = 0.01; OR = 0.06; 95% CI = 0.02–0.11), NLR (p = 0.018; OR = 0.24; 95% CI = 0.05–0.44), and complement 3 (C3) (p < 0.001; OR = 7.24; 95% CI = 3.77–10.72) were independent and significant risk factors for predicting the severity of disability (as assessed by the EDSS score) during the acute phase. Further ROC curve analysis demonstrated that C3 had strong predictive performance (AUC = 0.84), with high sensitivity and specificity at the optimal cut-off value. NLR showed limited overall discrimination (AUC = 0.62) but high specificity, suggesting a potential role as a rule-in marker. Age exhibited modest predictive value (AUC = 0.64), with an optimal cut-off of 45.5 years.

**Conclusion:**

Older age at onset, a higher NLR, and elevated serum C3 levels may predict greater disability in AQP4-IgG-positive NMOSD patients during the acute phase. Early identification of these predictive indicators could help guide initial treatment decisions.

## 1 Introduction

Neuromyelitis optica spectrum disorder (NMOSD) is an autoimmune-mediated inflammatory demyelinating condition that primarily targets the optic nerves, spinal cord and certain regions of the brain within the central nervous system (CNS) (1, 2). The identification of the specific autoantibody aquaporin-4 immunoglobulin G (AQP4-IgG) has transformed our approach to NMOSD, enabling precise diagnosis and clarifying its pathogenesis apart from that of other demyelinating diseases such as multiple sclerosis (MS) (3). The binding of AQP4-IgG to astrocytic AQP4 triggers complement activation and infiltrates granulocytes, eosinophils, and lymphocytes, ultimately causing astrocyte damage, oligodendrocyte injury, demyelination, and neuronal death (4). However, approximately 20% to 30% of NMOSD patients are AQP4-IgG seronegative, with ongoing controversy surrounding whether seropositive and seronegative forms are part of the same disease spectrum (5, 6).

Despite advances in serological testing and the development of targeted therapies, NMOSD remains a highly disabling condition, with a significant proportion of patients sustaining permanent visual, motor, or sensory deficits following acute attacks. The acute phase is particularly critical, as early and aggressive intervention is essential to mitigate long-term disability. However, the identification of patients at greatest risk of severe disability during an acute attack remains challenging. Consequently, there is a pressing need to identify clinically feasible and economically efficient predictors of disability progression that can be readily integrated into routine practice.

Several studies have sought to identify predictors of disability in NMOSD, focusing on demographic, clinical, and immunological factors. For instance, younger age at onset has been associated with a higher risk of visual disability in some cohorts (7), while other studies suggest that older age may predict worse motor outcomes (8). Racial differences, onset phenotypes (e.g., optic neuritis or cerebral syndrome), and response to acute therapies have also been implicated in long-term disability (9).

Inflammatory biomarkers—such as the neutrophil-to-lymphocyte ratio (NLR), platelet-to-lymphocyte ratio (PLR), monocyte-to-lymphocyte ratio (MLR), systemic inflammation response index (SIRI; monocyte × NLR), and systemic immune inflammation index (SII; platelet × NLR)—have emerged as potential indicators of disease activity and severity in various autoimmune conditions (10–13). Prior studies have indicated that NLR is significantly elevated during acute attacks and throughout the follow-up period in NMOSD, yet it does not serve as an independent predictor of clinical or radiographic outcomes (14). Conversely, a high PLR is recognized as a hallmark of active NMOSD, while an elevated monocyte-to-lymphocyte ratio MLR is considered a risk factor for disease relapse (15). Nevertheless, the relationship between these inflammatory markers and the severity of NMOSD in the acute phase remains poorly investigated.

Additionally, complement activation plays a well-established role in the pathogenesis of AQP4-IgG-positive NMOSD, contributing to blood-brain barrier disruption and astrocyte damage (16, 17). Notably, the complement 5 (C5) inhibitor eculizumab has demonstrated significant clinical efficacy in preventing relapses in NMOSD (18). Previous studies have demonstrated the involvement of complement components, particularly complement 3 (C3) and complement 4 (C4), in patients with NMOSD. Most evidence suggests reduced complement levels, likely reflecting complement activation and consumption during disease activity, although some variability across studies still exists (19, 20). Their potential role in predicting acute disease severity warrants further investigation.

In this prospective cohort study, we aimed to identify clinical and laboratory predictors of acute-phase disability in AQP4-IgG-positive NMOSD patients. Our findings may help guide initial treatment decisions and improve acute management strategies for this devastating disease.

## 2 Materials and methods

### 2.1 Participants

We prospectively recruited patients diagnosed with NMOSD from the Department of Neurology, Guangdong Provincial Hospital of Chinese Medicine, between April 2022 and June 2025. The diagnosis was established in accordance with the 2015 International Panel for NMO Diagnosis (IPND) criteria (1). Included patients had no history of antecedent infections within two weeks prior to hospitalization. Patients who were AQP4-IgG seronegative or had not been tested were excluded. Additionally, we excluded those with comorbid malignancies, rheumatoid arthritis, or other conditions requiring ongoing immunosuppressive therapy, as well as those previously treated with complement inhibitors or had recurrent hospitalizations. Furthermore, 25 healthy controls matched for sex and age were enrolled in the study.

The study was approved by the Ethics Committee of Guangdong Provincial Hospital of Chinese Medicine (Approval ID: ZE2022-062-01), and all participants provided written informed consent.

### 2.2 Laboratory measurements

Blood samples were collected under fasting conditions after hospital admission and prior to the initiation of any therapeutic interventions. Blood samples were processed according to standard clinical laboratory procedures. As part of routine clinical care, routine hematological and biochemical parameters were measured immediately after collection. For specific biomarkers such as neurofilament light chain (NfL) and glial fibrillary acidic protein (GFAP), samples were centrifuged, aliquoted, and stored at -80°C until batch analysis.

### 2.3 Data collection

We collected demographic data including age, sex, history of attacks, and medication use. Laboratory parameters consisted of complete blood cell count (CBC), interleukin-6 (IL-6), 25-hydroxyvitamin D [25(OH)D], C3, C4, immunoglobulin (Ig), GFAP and NfL. Imaging data included the number of spinal cord segments involved. The NLR, PLR, MLR, SII and SIRI were derived from peripheral blood cell counts. Disability was assessed using the Expanded Disability Status Scale (EDSS) score (21), as evaluated by two certified neurologists.

### 2.4 Statistical analysis

Data processing and analysis were performed using R version 4.3.3 (2024-02-29). Continuous variables were expressed as mean ± standard deviation (SD) or median with interquartile range (IQR; 25%–75%), while categorical variables are presented as numbers and percentages. Categorical variables were compared using the Chi-square test. For continuous variables, parametric tests were applied for normally distributed data, and nonparametric tests were used for non-normally distributed data. Associations between predictive factors and EDSS scores were examined using both univariate and multivariate linear regression models. A p-value of less than 0.05 was considered statistically significant.

## 3 Results

### 3.1 Demographic and clinical characteristics

**Figure 1:**
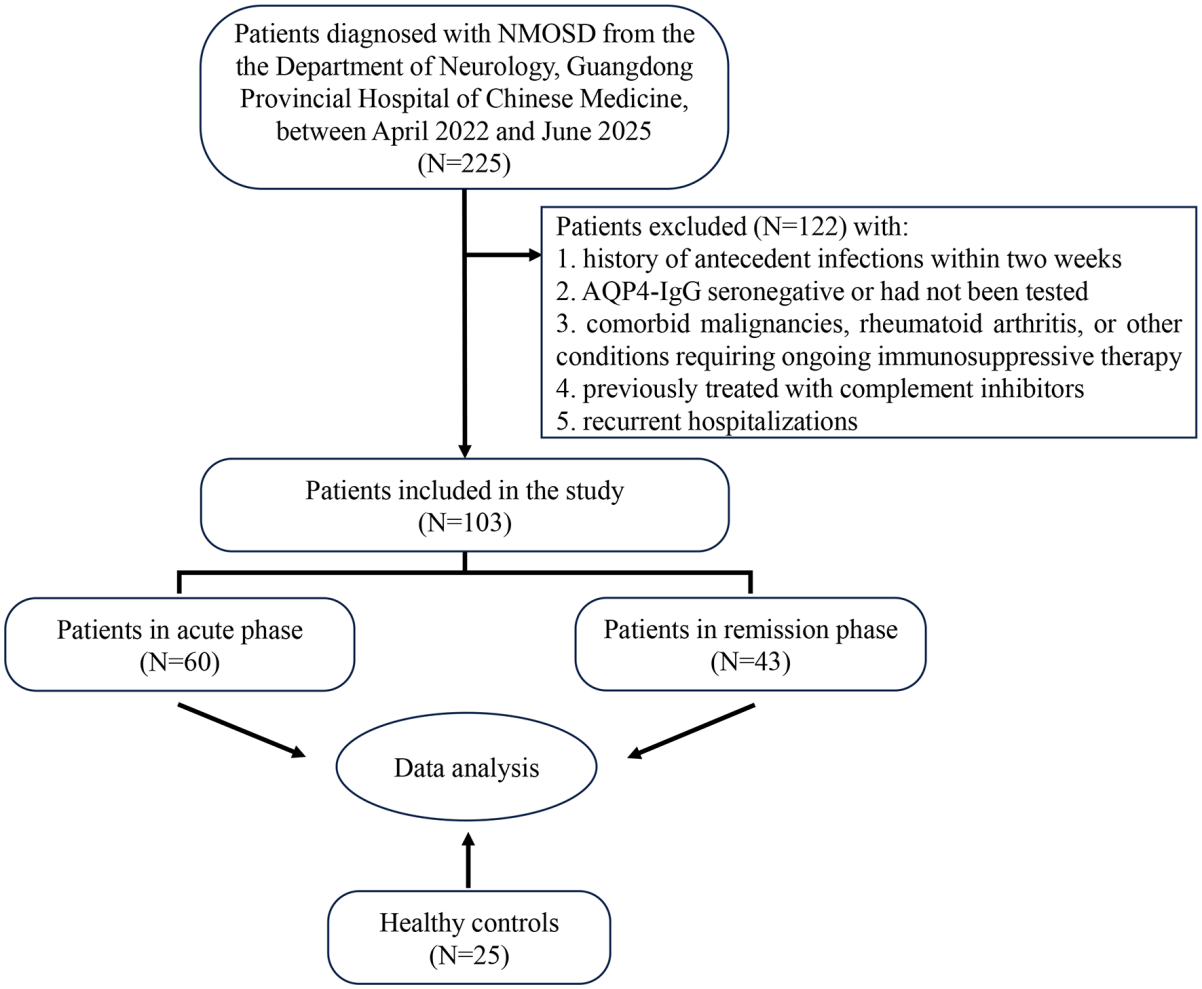
Flow chart.

A total of 225 patients with NMOSD were screened in this study. Ultimately, 103 eligible NMOSD patients were enrolled (60 in acute phase and 43 in remission), along with 25 sex- and age-matched healthy subjects who were also included in the analysis. Among the 60 acute-phase patients, 16 were experiencing their disease onset and 44 were in relapse. The acute phase is defined as new or recurrent neurological symptoms lasting more than 24 hours, without identifiable causes such as fever, infection, or other autoimmune diseases, with symptoms persisting or worsening from disease onset until admission, and typically occurring more than 30 days after the last attack (22). The specific procedures are illustrated in **Figure 1**.

**Table 1** presents the comparison of clinical measurements among acute-phase patients, remission-phase patients, and healthy controls. Preliminary data revealed statistically significant differences among the three groups in terms of age, EDSS scores, white blood cell (WBC) count, neutrophil (NEUT) count, NLR, MLR, SII, Siri, IgA, IgG, and C4 levels (P<0.05).

**Table 1:** Demographic and clinical characteristics of participants.

Characteristic	Total (n = 128)	Remission NMOSD (n = 43)	Acute NMOSD 1 (n = 60)	HCs (n = 25)	P value	P1 value	P2 value	P3 value
Sex; n(%)					0.831^	–	–	–
Male	10 (7.81)	4 (9.30)	5 (8.33)	1 (4.00)				
Female	118 (92.19)	39 (90.70)	55 (91.67)	24 (96.00)				
Age; Mean ± SD	46.81 ± 12.63	50.93 ± 13.65	44.45 ± 12.67	45.40 ± 8.79	**0.029**△	**0.027**	0.182	0.944
EDSS; M (Q1; Q3)	3.00 (1.50; 4.50)	2.50 (1.25,3.50)	3.50 (2.00,6.50)	–	**0.027^Z^**	–	–	–
Attacks; M (Q1; Q3)	1.00 (0.00; 3.00)	2.00 (1.00,3.00)	1.00 (0.00,2.00)	–	0.176^Z^	–	–	–
WBC; M (Q1; Q3)	6.11 (4.88; 8.48)	6.37 (4.93,7.78)	6.54 (5.44,9.65)	4.93 (4.32,5.84)	**<.001** **^#^**	0.318	**0.005**	**<0.001**
NEUT; M (Q1; Q3)	3.81 (2.88; 5.42)	3.85 (3.05,5.17)	4.56 (3.69,7.16)	2.66 (2.30,3.46)	**<.001^#^**	0.071	**<0.001**	**<0.001**
NLR; M (Q1; Q3)	2.29 (1.72; 3.56)	2.17 (1.63,3.32)	2.73 (1.99,4.37)	1.67 (1.13,2.02)	**<.001^#^**	**0.031**	**0.002**	**<0.001**
PLR; M (Q1; Q3)	145.09 (113.13; 196.80)	134.74 (108.29,182.27)	163.61 (121.84,215.15)	145.07 (104.87,183.05)	0.371^#^	–	–	–
MLR; M (Q1; Q3)	0.22 (0.17; 0.30)	0.24 (0.20,0.31)	0.23 (0.17,0.32)	0.16 (0.12,0.21)	**<.001^#^**	**0.297**	**<0.001**	**<0.001**
SII; M (Q1; Q3)	580.98 (411.25; 907.67)	535.96 (398.87,908.44)	709.61 (537.09,1012.74)	405.58 (259.78,523.33)	**<.001^#^**	**0.021**	**0.007**	**<0.001**
SIRI; M (Q1; Q3)	0.89 (0.56; 1.41)	1.03 (0.70,1.60)	1.10 (0.72,1.53)	0.47 (0.32,0.59)	**<.001^#^**	0.810	**<0.001**	**<0.001**
IL-6; M (Q1; Q3)	2.27 (0.75; 4.16)	2.80 (1.81,4.73)	2.04 (0.75,4.45)	2.25 (1.77,3.05)	0.215^#^	–	–	–
IgA; M (Q1; Q3)	2.13 (1.66; 2.65)	1.67 (1.34,2.18)	2.18 (1.73,3.04)	2.26 (1.78,2.55)	**0.004^#^**	**0.003**	**0.005**	0.751
IgG; M (Q1; Q3)	12.10 (8.84; 14.30)	9.25 (7.02,11.30)	12.60 (9.50,14.30)	14.00 (11.90,14.90)	**<.001^#^**	**0.004**	**<0.001**	0.069
IgM; M (Q1; Q3)	0.93 (0.64; 1.36)	0.78 (0.41,1.25)	1.01 (0.66,1.38)	1.04 (0.83,1.27)	0.167^#^	–	–	–
C3; M (Q1; Q3)	1.06 (0.94; 1.16)	1.06 (0.85,1.17)	1.04 (0.94,1.16)	1.15 (0.98,1.19)	0.235^#^	–	–	–
C4; M (Q1; Q3)	0.22 (0.17; 0.28)	0.19 (0.15,0.25)	0.21 (0.17,0.28)	0.27 (0.23,0.32)	**<.001^#^**	0.311	**<0.001**	**0.001**
25(OH)D; M (Q1; Q3)	51.70 (39.92; 68.18)	55.70 (48.60,70.50)	49.80 (39.00,64.70)	–	0.271^Z^	–	–	–
GFAP; M (Q1; Q3)	85.32 (62.74; 140.46)	80.04 (66.20,87.83)	94.00 (59.34,191.69)	–	0.331^Z^	–	–	–
Nfl; M (Q1; Q3)	13.28 (6.60; 26.00)	8.05 (5.70,13.77)	17.29 (7.30,53.43)	–	0.078^Z^	–	–	–

△, ANOVA; #, Kruskal-waills test; ^, Fisher exact; Z, Mann-Whitney test.

SD, standard deviation; M, Median; Q_1_, 1st Quartile; Q_3_, 3st Quartile.

*P1, Comparison results between the remission phase group and the acute phase group; P2, comparison results between the remission phase group and HC group; P3, comparison results between the acute phase group and HC group [using *post-hoc* test (Dunn's test or Tukey's HSD test)].

Bold values indicate P < 0.05.

*Post-hoc* analysis revealed that patients in the remission phase were generally older compared to those in the acute phase group. WBC count, NEUT count, NLR, MLR, SII and SIRI were significantly higher in both the acute and remission groups than in the HC group, whereas serum C4 levels were lower (P < 0.05). Additionally, patients in the acute phase exhibited significantly higher levels of NLR, SII, IgA, and IgG compared to those in the remission phase (P < 0.05).

### 3.2 Univariate linear regression analysis

Based on the above findings, to further investigate the predictors of disability severity during the acute phase, we focused specifically on patients in the acute stage as the primary study population. Univariate linear regression was performed to evaluate the association between various clinical and laboratory factors and the severity of acute AQP4-IgG-seropositive NMOSD. The results are summarized in **Table 2**.

**Table 2:** Univariate analysis of disability severity among patients with acute NMOSD.

Variables	β	S.E	t	P value	β (95%CI)
First attack					
No					0.00 (Reference)
Yes	-0.76	0.74	-1.04	0.305	-0.76 (-2.21 ~ 0.68)
Age	0.07	0.03	2.61	**0.012**	0.07 (0.02 ~ 0.12)
Number of episodes	-0.01	0.20	-0.07	0.944	-0.01 (-0.40 ~ 0.38)
WBC	0.05	0.12	0.45	0.655	0.05 (-0.17 ~ 0.28)
NEUT	0.12	0.14	0.81	0.419	0.12 (-0.16 ~ 0.39)
NLR	0.28	0.11	2.48	**0.016**	0.28 (0.06 ~ 0.51)
MLR	2.44	1.24	1.97	0.054	2.44 (0.01 ~ 4.87)
SII	0.01	0.00	2.30	**0.025**	0.01 (0.01 ~ 0.01)
SIRI	0.35	0.15	2.31	**0.025**	0.35 (0.05 ~ 0.64)
IL-6	0.06	0.03	2.19	**0.033**	0.06 (0.01 ~ 0.12)
IgA	0.36	0.38	0.94	0.349	0.36 (-0.39 ~ 1.11)
IgG	0.08	0.06	1.22	0.229	0.08 (-0.05 ~ 0.20)
C3	8.03	1.79	4.48	**<0.001**	8.03 (4.52 ~ 11.54)
C4	7.44	4.21	1.77	0.083	7.44 (-0.81 ~ 15.69)
Spinal cord segments involved	0.16	0.05	2.96	**0.004**	0.16 (0.05 ~ 0.26)

CI, Confidence Interval.

Bold values indicate P < 0.05.

Several factors were significantly associated with disease severity. These included age (β = 0.07, 95% CI: 0.02-0.12, P = 0.012), NLR (β = 0.28, 95%CI: 0.06-0.51, P = 0.016), SII (β = 0.01, 95% CI: 0.01-0.01, P = 0.025), SIRI (β = 0.35, 95% CI: 0.05-0.64, P = 0.025), IL-6 (β = 0.06, 95% CI: 0.01-0.12, P = 0.033), C3 (β = 8.03, 95% CI: 4.52-11.54, P < 0.001) and the number of involved spinal cord segments (β = 0.16, 95% CI: 0.05-0.26, P = 0.004). Factors such as NEUT, C4, number of episodes did not show significant associations (P> 0.05).

### 3.3 Multivariable linear regression analysis

Variables with P-value less than 0.05 in the univariate analysis were included in the multivariable linear regression model to identify independent predictors of disease severity. The results are presented in **Table 3**.

**Table 3:** Multivariate analysis of disability severity among patients with acute NMOSD.

Variables	β	S.E	t	P value	β (95%CI)
Age	0.06	0.02	2.72	0.010	0.06 (0.02 ~ 0.11)
NLR	0.24	0.10	2.45	0.018	0.24 (0.05 ~ 0.44)
C3	7.24	1.77	4.08	<0.001	7.24 (3.77 ~ 10.72)
Spinal cord segments involved	0.10	0.06	1.73	0.091	0.10 (-0.01 ~ 0.21)

CI, Confidence Interva.l

Bold values indicate P < 0.05.

After adjustment, age (β = 0.06, 95% CI: 0.02-0.11, P = 0.010), NLR (β = 0.24, 95% CI: 0.05-0.44, P = 0.018), and C3 (β = 7.24, 95% CI: 3.77-10.72, P < 0.001) remained significantly associated with disease severity. The number of involved spinal cord segments showed a trend toward significance but did not retain it in the multivariable model (β = 0.10, 95% CI: -0.01-0.21, P = 0.091).

### 3.4 ROC curve analysis for prediction of severe disability

**Figure 2:**
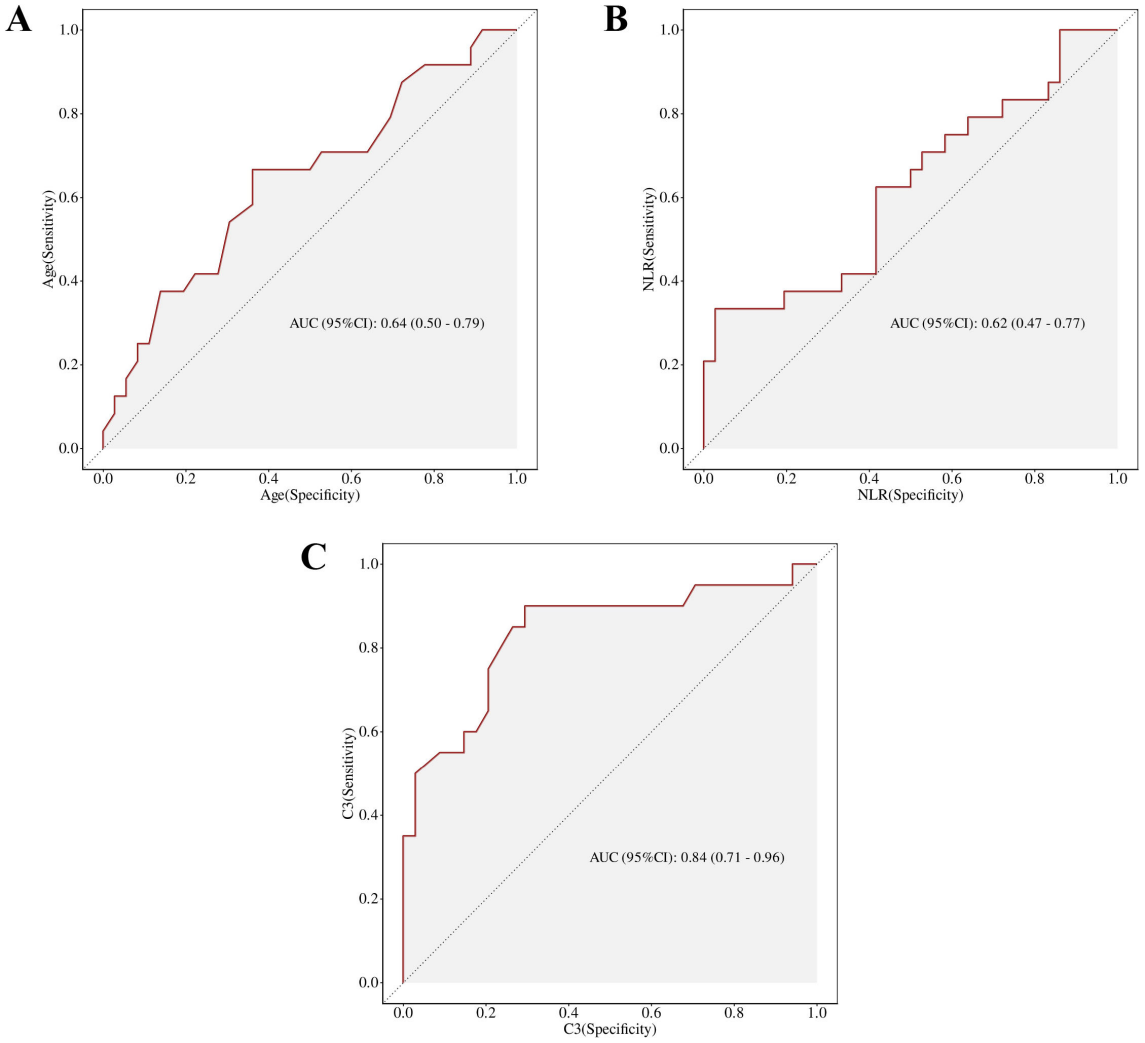
ROC curve analysis of age, NLR, and C3 for predicting severe disability (EDSS ≥ 4) in acute NMOSD.

To further evaluate the clinical predictive value of these variables, receiver operating characteristic (ROC) curve analysis was performed using EDSS ≥ 4 as the definition of severe disability (**Figure 2**). The optimal cut-off values for continuous variables were determined based on the maximum Youden index.

The optimal thresholds were 45.5 years for age, 5.449 for NLR, and 1.025 for C3. Among the evaluated variables, C3 demonstrated the strongest discriminatory ability, with an AUC of 0.84 (95% CI: 0.64–0.88), a sensitivity of 0.90 (95% CI: 0.77–1.00), and a specificity of 0.71 (95% CI: 0.55–0.86) at the optimal cut-off value. NLR showed a relatively low overall predictive performance, with an AUC of 0.62(95% CI: 0.47–0.77). At the optimal threshold of 5.449, it yielded a low sensitivity (0.33, [95% CI: 0.14–0.52]) but a very high specificity (0.97, [95% CI: 0.92–1.00]), indicating strong rule-in capability despite limited screening performance. Age exhibited modest predictive ability, with an AUC of 0.64 (95% CI: 0.50–0.79). Using the optimal cut-off value of 45.5 years, the sensitivity and specificity were 0.67 (95% CI: 0.48–0.86) and 0.64 (95% CI: 0.48–0.80), respectively.

## 4 Discussion

This study investigated the impact of multiple factors on the severity of disability (as measured by the EDSS score) in AQP4-IgG seropositive NMOSD patients during the acute phase. Our results demonstrated that age at onset, NLR, and C3 level were independent and significant predictors of acute-phase disability severity (EDSS score). Importantly, ROC analysis further clarified their clinical predictive performance, revealing distinct roles for each biomarker.

The findings demonstrated that advanced age was an independent risk factor for the severity of disability in the acute phase of NMOSD, while its predictive performance was modest (AUC = 0.64). The identified cut-off value of 45.5 years suggested that patients above this threshold may be at increased risk of severe disability. Prior research placed greater emphasis on the impact of age on disease prognosis. A multicenter cohort study (8) from the United Kingdom and Japan demonstrated that onset age was a key independent risk factor for predicting disability outcomes—particularly motor dysfunction and mortality—in patients with NMOSD, with advanced age being associated with a poorer prognosis. Another study from South Korea (23) revealed a significant positive correlation between age at onset and the EDSS score at the last follow-up. Furthermore, a study conducted in France (24) indicated that patients with late-onset NMOSD experienced more severe initial attacks, were more prone to significant motor impairment, and faced a higher risk of mortality, which was consistent with our findings. Advanced age compromises the intrinsic regenerative capacity of the central nervous system (CNS)—manifested as impaired remyelination, axonal regeneration, and diminished neurotrophic support—thereby increasing its vulnerability to acute inflammatory damage (25–27). Furthermore, age-induced immunosenescence alters inflammatory response patterns, which can exacerbate tissue injury and dysregulate immune resolution (28, 29). Therefore, acute attacks in elderly patients require closer monitoring and more aggressive therapeutic management.

In our study, systemic inflammatory indices (NLR, MLR, SII, SIRI, IL-6) were all positively correlated with EDSS scores, indicating that the intensity of the systemic inflammatory response during the acute phase was closely associated with the severity of neurological damage. After adjustment, NLR emerged as an independent and significant predictor. NLR showed relatively limited overall discriminatory ability (AUC = 0.62). However, its extremely high specificity (0.97) indicates that elevated NLR levels may strongly suggest severe disease. NLR had been extensively investigated in various autoimmune diseases and had recently gained increasing attention in demyelinating disorders of the CNS. In MS, several studies demonstrated that NLR values were higher in patients than in healthy controls (30–33). Some reports indicated a significant association between elevated NLR and increased disability in MS patients, and relapse phases were shown to exhibit higher NLR levels than remission phases. However, the role of NLR in predicting disability progression remained controversial. In myelin oligodendrocyte glycoprotein antibody-associated disease (MOGAD), elevated NLR was observed during acute attacks and was suggested to aid in distinguishing acute relapses from remission (34). Regarding NMOSD, several studies had reported a positive correlation between NLR and EDSS scores (15, 35–38). Nevertheless, other research found (14) that a high NLR was not an independent predictor of clinical or radiological outcomes in AQP4-IgG-positive NMOSD patients. Our study specifically focused on AQP4-IgG-positive NMOSD patients in the acute phase, and our model identified serum NLR as an independent and significant predictor of disability severity. This implied that patients with elevated NLR might require more aggressive and potent treatment strategies to promptly control inflammation and maximize the preservation of neurological function.

Another key finding of this study was that serum C3 could serve as a critical biomarker for predicting the degree of disability during the acute phase in AQP4-IgG-positive NMOSD. Among these variables, C3 demonstrated the strongest predictive ability, with an AUC of 0.84. Complement activation plays a well-established role in the pathophysiology of NMOSD (16, 17), our finding underscores the central role of complement activation in NMOSD pathophysiology. As the central component of the complement cascade, the serum level of C3 was found to reflect, to some extent, the magnitude of complement-dependent cytotoxicity (CDC) and the ensuing destruction of astrocytes. Previous studies had demonstrated that serum C3 levels were significantly lower in patients with acute-phase NMOSD compared to both healthy controls and the MOGAD group (39, 40), while levels in patients in remission could be either reduced or unchanged (19,^20^). In contrast, our study did not show a statistically significant difference in C3 levels among the acute-phase, remission-phase, and healthy control groups. The inconsistency in results could be attributed to factors such as sample size, differences in detection techniques, or the duration of the disease. Furthermore, our results indicated that, the high sensitivity of C3 suggested that it may serve as an effective early indicator for identifying patients at high risk of severe disability, supporting timely and aggressive therapeutic intervention. This finding was highly consistent with the known pathological mechanism of AQP4-IgG-positive NMOSD and strongly supported the use of complement inhibition as a therapeutic strategy.

The number of previous attacks did not demonstrate a significant association with the EDSS scores in this study (p = 0.944). This might suggest that the severity of the current attack is governed more by factors active during the acute phase (such as inflammation and complement activation) than by the mere accumulation of past episodes.

From a clinical perspective, these findings suggested a potential framework for early risk assessment in acute NMOSD. Elevated C3 levels may identify high-risk patients requiring aggressive intervention, while markedly increased NLR may reinforce the likelihood of severe disease. Age may further refine risk stratification, particularly in older patients.

This study had several limitations. First, it was a single-center investigation, which limited the generalizability of the findings to broader populations. Therefore, larger multi-center cohort studies were required to further investigate these observations in the future. Second, the study did not evaluate the long-term outcomes of the patients; thus, well-designed follow-up protocols were suggested for future research to explore the predictive value of the aforementioned indicators for prognosis.

## 5 Conclusions

Our study indicated that older age at onset, a higher NLR, and elevated serum C3 levels may predict greater disability in AQP4-IgG-positive NMOSD patients during the acute phase. Early identification of these predictive indicators could help guide initial treatment decisions.

## Data Availability

The raw data supporting the conclusions of this article will be made available by the authors, without undue reservation.
